# Known Allergen Structures Predict *Schistosoma mansoni* IgE-Binding Antigens in Human Infection

**DOI:** 10.3389/fimmu.2015.00026

**Published:** 2015-02-03

**Authors:** Edward J. Farnell, Nidhi Tyagi, Stephanie Ryan, Iain W. Chalmers, Angela Pinot de Moira, Frances M. Jones, Jakub Wawrzyniak, Colin M. Fitzsimmons, Edridah M. Tukahebwa, Nicholas Furnham, Rick M. Maizels, David W. Dunne

**Affiliations:** ^1^Department of Pathology, University of Cambridge, Cambridge, UK; ^2^European Bioinformatics Institute, Cambridge, UK; ^3^Institute of Immunology and Infection Research, The University of Edinburgh, Edinburgh, UK; ^4^Institute of Biological, Environmental and Rural Sciences, Aberystwyth University, Aberystwyth, UK; ^5^Vector Control Division, Ugandan Ministry of Health, Kampala, Uganda; ^6^Department of Pathogen Molecular Biology, London School of Hygiene and Tropical Medicine, London, UK

**Keywords:** schistosomiasis mansoni, allergenicity, IgE, IgG4, parasite allergens, metazoan parasite, helminth proteins

## Abstract

The IgE response has been associated with both allergic reactions and immunity to metazoan parasites. Recently, we hypothesized that all environmental allergens bear structural homology to IgE-binding antigens from metazoan parasites and that this homology defines the relatively small number of protein families containing allergenic targets. In this study, known allergen structures (Pfam domains) from major environmental allergen families were used to predict allergen-like (SmProfilin, SmVAL-6, SmLipocalin, SmHSP20, Sm triosephosphate isomerase, SmThioredoxin, Sm superoxide dismutase, SmCyclophilin, and Sm phosphoglycerate kinase) and non-allergen-like [Sm dynein light chain (SmDLC), SmAldolase SmAK, SmUbiquitin, and Sm14-3-3] proteins in *Schistosoma mansoni*. Recombinant antigens were produced in *Escherichia coli* and IgG1, IgG4, and IgE responses against them measured in a cohort of people (*n* = 222) infected with *S. mansoni*. All allergen-like antigens were targeted by IgE responses in infected subjects, whilst IgE responses to the non-allergen-like antigens, SmAK, SmUbiquitin, and Sm14-3-3 were essentially absent being of both low prevalence and magnitude. Two new IgE-binding Pfam domain families, not previously described in allergen family databases, were also found, with prevalent IgE responses against SmDLC (PF01221) and SmAldolase (PF00274). Finally, it was demonstrated that immunoregulatory serological processes typically associated with allergens also occurred in responses to allergen-like proteins in *S. mansoni* infections, including the production of IgG4 in people responding with IgE and the down-regulation of IgE in response to increased antigen exposure from *S. mansoni* eggs. This study establishes that structures of known allergens can be used to predict IgE responses against homologous parasite allergen-like molecules (parallergens) and that serological responses with IgE/IgG4 to parallergens mirror those seen against allergens, supporting our hypothesis that allergenicity is rooted in expression of certain protein domain families in metazoan parasites.

## Introduction

The role of IgE in allergic immune responses is well established and extensive research has characterized ≈3000 known allergens (1, 2). Further analysis of known allergens has shown that they occupy a highly restricted set of 255 protein domains, defined in the Pfam database by their structure and function, representing <2% of all known protein domain families (3). Furthermore just 10 Pfam protein domains represent no fewer than 40% of the 995 molecular protein allergens described in the Allfam allergen family database. These “top 10” families containing the greatest number of different individual allergenic proteins are (in order of frequency) Prolamin, EF-hand, Tropomyosin, CAP (CRISP/Antigen-5/PR-1), Subtilisin-like serine proteases, Lipocalin, Profilin, Bet v 1, Expansin, and Cupin (3). The reasons for the predominance of a restricted molecular subset and the principles underlying “What makes an allergen an allergen?” are currently not known. Many different biochemical properties of proteins have been suggested to be determinants of allergenicity including size, solubility, hydrophobicity, protein fold stability, oligermerization, venom toxicity, post-translational modifications, and protease activity (2, 4). However, none of these factors independently describe all allergens or indeed differentiate satisfactorily between allergens and non-allergens, for example, not all allergens are proteases and there are many examples of non-allergenic proteases.

We have therefore suggested a new paradigm, which defines a common characteristic of potentially allergenic structural protein families, and explains why they are restricted in number (5). Potent IgE-mediated immune responses are seen during infections with metazoan parasites, including both helminth (worm) endoparasites and ectoparasites such as arthropod ticks. Moreover, there is a growing body of evidence to suggest that the IgE-mediated immune response, which is a recently evolved immune mechanism found only in mammals, helps protect against infection with metazoan parasites (6–13). Furthermore, given that metazoan parasites have similar cell biology to the metazoan host, it would be expected that potential targets for antibody responses would exclude proteins with high identity to their equivalents in the host. This appears to be more marked with IgE than other antibody isotypes and most environmental allergens share <70% identity with their human equivalents (14).

Examination of the “top 10” allergen families in the context of the human antibody responses against infecting metazoan parasites revealed examples of parasite proteins with known IgE-binding domains or “parallergens” in three allergen families; EF-hand domains in Tegumental-Allergen-Like (TAL) proteins from schistosomes (15), Tropomyosin proteins in filarial nematodes and schistosomes (16, 17), and CAP protein domains in hookworm nematodes (18). A further five families; Subtilisin-like serine protease, Lipocalin, Profilin, Bet v 1, and Cupin families, contain metazoan parasite homologs with the potential to be targeted by IgE during human parasitic infections (5). Additionally there are many known immunogenic proteins, with potential IgE-binding activity, within the most abundant schistosome proteins that may have homology to known allergens (19–22).

When attempting to identify IgE responses to parasite proteins it is important to consider the effects of chronic helminth infection in humans, and the shifts between IgE and IgG4 isotypes that occur during long-term exposure (23). Responses to parallergens in helminth-infected subjects may result in the down-regulation of IgE antibodies and the production of IgG4 to the same allergenic epitopes (15, 24). This shift is well-documented in allergy, especially in specific immunotherapy (SIT), where constant low-level exposure to a specific allergen results in increased IgG4 and decreased IgE levels (25, 26). In individuals with schistosomiasis mansoni, the expression of proteins across the *S. mansoni* life cycle and their localization in specific tissues may be important in determining the levels of exposure of a protein to the immune system and the corresponding antibody responses. The human life cycle stages of *S. mansoni* include infective skin-penetrating cercaria, host tissue-migrating schistosomule, blood-dwelling adult worms, and eggs (27). Due to a number of immune evasion mechanisms, protein antigens in cercaria, schistosomules, and adult worms are generally sequestered away from immune system and are only exposed upon the death of the adult worms, which may take between 3 and 10 years (28). On the other hand, antigens present in eggs (thousands of which are released daily) are continuously exposed to the immune system during the death of eggs that have failed to escape the host and become trapped in tissues, a process, which usually takes 1–2 weeks. Indeed studies in schistosomiasis mansoni endemic areas on the SmTAL family of EF-hand parallergens have shown that for antigens expressed exclusively in the adult worm stage of the parasite, such as SmTAL1 and SmTAL3, IgE responses only develop naturally over time as adult worms die or during treatment with praziquantel (PZQ), which damages adult worms and reveals cryptic antigens (24). Conversely, for antigens expressed strongly in eggs, such as SmTAL2, IgE antibodies are seen only during early infection (children <5 years) with IgE responses down regulated and replaced with IgG4 antibody responses in older individuals (24, 29). Therefore in determining the “allergen-like” properties of *S. mansoni* predicted parallergens it is important to consider their expression in eggs/adult worms, as well as both IgE and IgG4 antibody levels to each antigen.

We therefore hypothesize that known IgE-binding domains may be used to predict the protein targets of allergy-like responses in individuals infected with *S. mansoni*, including targeting by IgE, the induction of regulatory responses such as targeting by IgG4 and the down-regulation of IgE in response to continuous exposure of antigens during infection. In this study, we now show that known allergen structures can indeed be used to predict IgE-binding and allergy-like responses against recombinant *S. mansoni* parallergens in an infected human cohort from a schistosomiasis mansoni endemic area of Uganda.

## Materials and Methods

### Parasite material

A Puerto Rican strain of *S. mansoni* was used in this study. RNA was isolated and extracted from cercaria and adult worms as previously described (30). RNA integrity was verified using a Bioanalyser (Agilent Technologies, Bracknell, UK). cDNA was prepared from 1 μg total RNA using random hexamers (Sigma-Aldrich, Gillingham, UK) and Superscript II reverse transcriptase according to the manufacturer’s instructions (Life Technologies, Paisley, UK).

### Bioinformatic analysis

Hidden Markov Models (HMMs) of Pfam domains associated with the “top 10” allergen families listed in Allfam^1^ were used to search the Uniprot database^2^ to identify any *S. mansoni* proteins containing the relevant Pfam domains (3, 31). Similarly Pfam domains associated with *S. mansoni* proteins abundant in human life cycle stages [as identified by proteomics (21)] were used to search Allfam for functional sequence similarity to families of known allergens.

To compare known parasite and allergen structures, protein sequences of *S. mansoni* proteins with known 3D structure were used to search Allergome^3^ for allergens with similar sequence. Allergens with an *E*-value cut-off of <1 × 10^−4^ were searched for corresponding known 3D structures in Protein Data Bank^4^. Protein models were generated using MODELLER v9.10 (32) and LSQMAN^5^ was used to perform protein structural alignments by least squares super-positioning with the quality of the models assessed by root mean squared deviation (RMSD).

### Molecular cloning

SmTAL1 (GenBank M37003.1), SmTAL2 (GenBank M67506.1), Sm triosephosphate isomerase (SmTPI) (GenBank M83294.1), and SmTropomyosin II (GenBank KC904504.1) were produced using the pGEX family of plasmids during other studies (17, 24, 33). pGEX-KG plasmid containing Sm dynein light chain (SmDLC) (GenBank U55992.1) was a kind gift from Karl Hoffmann (University of Aberystwyth). The SmVAL-6 CAP domain (amino acids 1–145 of full-length SmVAL-6, GenBank AY953433) was cloned into the pET-30a vector. All other antigens were cloned in the pGEX-KG vector. Gene sequences were derived from GenBank and primers containing unique restriction enzymes sites were designed using Primer3Plus (Table S1 in Supplementary Material)^6^. PCR to generate gene specific coding sequences was performed using Phusion High Fidelity DNA polymerase (Thermo Fisher Scientific, Reading, UK) in a Tetrad 2 thermal cycler (MJ research/Bio-Rad, Hemel Hempstead, UK). PCR products and plasmids were digested with the relevant pair of FastDigest restriction enzymes (Table S1 in Supplementary Material). Additionally, plasmids were treated with FastAP (Thermo Fisher Scientific) to prevent self-ligation and ligation was performed using T4 DNA ligase (Thermo Fisher Scientific). Ligated plasmids were used to transform chemically competent DH5α *E. coli* cells by heat shock. Sanger sequencing was used to characterize cloned sequences. The sequences of SmLipocalin, Sm phosphoglycerate kinase (SmPGK), SmCyclophilin, and SmAldolase were found to be identical to previously cloned and sequenced cDNAs; GenBank Accession numbers M60895.1, L36833.1, L46884.1, and L38658.1. For molecules that had not been previously cloned and sequenced or differed from previous submissions, sequences were uploaded to GenBank with the following accession numbers; SmProfilin, KJ545485; Sm14-3-3, KM281669; SmUbiquitin, KM281670; SmAK, KM281671; SmThioredoxin, KM281672; Sm superoxide dismutase (SmSOD), KM281673; and SmHsp20, KM281674.

### Protein expression and purification

SmTAL1, SmTAL2, SmTPI, and SmTropomyosin II were expressed and purified as described previously (17, 24, 33). All other proteins ligated into pGEX-KG were expressed and purified as follows. Overnight cultures of TG2 *E. coli* containing the relevant plasmid were diluted 1:10, expanded to OD 0.4–0.8 and induced for 3–5 h with 1 mM IPTG. For SmSOD, TG2 *E. coli* cultures were also supplemented with 0.02 mM CuCl_2_ and 0.02 mM ZnCl_2_ and induced with 0.1 M IPTG as defined by Cardoso et al. (34). Cells were lysed using a French Pressure Cell at ≈10,000 psi and GST-fusion proteins isolated by affinity chromatography on Glutathione Sepharose 4B using an AKTAprime plus (GE Healthcare, Amersham, UK) as per the manufacturer’s instructions. Proteins were separated from their fusion partners by cleavage on Glutathione Sepharose using thrombin (GE Healthcare). Contaminating GST-fusion partner and thrombin were removed by incubation with Glutathione Sepharose 4B (GE Healthcare) and benzamadine-agarose beads (Sigma-Aldrich), respectively. SmVAL-6 was purified using a combination NiNTA and Q-Sepharose affinity chromatography as follows. Overnight cultures of BL21(DE3) *E. coli* containing the SmVAL-6 construct were cultured, induced, and lysed as above. Hexahistidine tagged proteins were isolated by affinity chromatography on NiNTA-agarose using an AKTAprime plus (GE Healthcare) as per the manufacturer’s instructions. Bacterial protein contamination was removed by Q-Sepharose ion exchange using a HiTrap Q (FF) column. Proteins were separated by elution on a 0–1 M NaCl gradient, and fractions containing only SmVAL-6 (120–350 mM NaCl) reserved. Proteins were tested for GST and bacterial contamination by in-house enzyme-linked immunosorbent assay (ELISA) assay using rabbit anti-GST antisera (Sigma) and rabbit anti-*E. coli* lysate antisera as previously described (24). Where required, MALDI fingerprinting was performed by the Protein and Nucleic Acid Chemistry facility (University of Cambridge Department of Biochemistry, Cambridge, UK).

### Study population

Venous blood samples were collected from volunteers living in the fishing village of Namoni, on the shores of Lake Victoria in the Mayuge district of South East Uganda. Transmission of schistosomiasis was consistently high throughout the year and the community received annual treatment as part of the Ugandan national control program. Three hundred seventy-two individuals (6–40 years, 57% female, mean age 18) with detectable infection (based on one stool sample, two Kato-Katz thick smears) were randomly selected from the village. Blood samples were collected, immediately prior to chemotherapy with PZQ (40 mg/kg body mass) and 5 weeks post-treatment, into heparinized tubes. Plasma was isolated by centrifugation at 2000 × *g* for 5 min, then aliquoted and stored at −80**°**C until use. Parasitological examinations were carried out before treatment and at 5 weeks post-treatment (to determine efficacy of treatment). For this study, we focused on 222 individuals (60% of total) for whom full serological and parasitological data were available (age 6–40 years, mean age 16 years, 59% female, pre-treatment prevalence of *S. mansoni* infection was 98.2%).

### Ethics statement

The Ugandan National Council of Science and Technology provided ethical clearance. Forms were translated in the local language and informed written assent was obtained from all adults and written consent from the parents/legal guardians of all children under 15.

### Enzyme-linked immunosorbent assay

Antigen-specific IgG1, IgG4, and IgE levels were measured in the plasma of infected individuals in duplicate by ELISA as described previously (24). The following modifications were made for specific antigens used in this protocol. Each antigen was applied to 384 well plates and incubated overnight at 4°C or dried to the plates at 37°C overnight as indicated in Table S2 in Supplementary Material. For each antigen, saturation coating concentrations and conditions were determined using an in-house competitive binding ELISA and were as shown in Table S2 in Supplementary Material. Human plasma was diluted 1:40 for IgE assays and 1:100 for IgG isotypes.

### Statistical analysis

Data management and statistical analysis was performed using STATA for Mac version 12.1. Change in the magnitude of antibody levels following treatment with PZQ were calculated as follows:

$$\%\;\mathrm{change}=100\times \left[e^{\bar{x}\left(\ln\left(\mathrm{AbMagnitudepost}-\mathrm{RX}+0.01\right)-\ln\left(\mathrm{AbMagnitudepre}-\mathrm{RX}+0.01\right)\right)}-1\right]$$

Significance in the changes of magnitudes were calculated by Wilcoxon matched-pairs signed-ranks test. Prevalences/proportions of responders were calculated following the application of assay sensitivity cut-offs (mean + 3σ) determined using sera from 26 normal European controls. Final cut-off values were determined following correction for outliers (NES responses greater than mean + 3σ) using in iterative process in which outliers were removed and the threshold recalculated until no outliers were found (Table S3 in Supplementary Material). Comparison of proportions of individuals responding with IgE alone vs. those responding with IgE and IgG4 together was performed by two-sample test of proportions. Significance in the difference in of antigen expression levels by microarray was calculated by one way ANOVA with Bonferroni post-tests for antigen-to-antigen differences. Correlations between IgE and egg expression levels were calculated using linear regression followed by Cook’s distance and leverage-vs.-squared-residual plot regression diagnostics to assess the impact of influential outliers. Regression diagnostics were performed to determine that assumptions were valid including, linearity, normal distribution of errors, and homoscedasticity of variance.

## Results

### Identification of allergen-like antigens in *S. mansoni*

Protein domain searches were used to identify *S. mansoni* proteins with functional domain sequence similarity (similar Pfam domains) to known allergens. Firstly, we searched for examples of *S. mansoni* proteins with Pfam domain similarity to allergens from the “top 10” Allfam allergen families. Proteins with similarity to the EF-Hand, Profilin, CAP, Tropomyosin, Lipocalin, and Subtilisin-like Serine Protease families listed in Allfam were found in *S. mansoni* (Table 1), whilst examples of proteins in the Bet v 1, Cupin, Expansin, and Prolamin families were not. Secondly, we searched for potential parallergens amongst the 32 *S. mansoni* proteins that were identified in the proteomic study of Curwen et al. (21) as being abundant in lifecycle stages found in the human host. Twenty-four (75%) were found to have domains with sequence similarity to known allergens (Table 2) whilst 8 (25%) did not.

**Table 1:** ***Schistosoma mansoni* antigens with functional sequence identity to abundant allergens**.

Protein	Pfam domain	Pfam name	Allergen family name (Allfam)	UniProt identifier
SmTAL1	PF00036	EF-Hand	EF-hand domain	Q4TTW6
SmProfilin	PF00235	Profilin	Profilin	G4VTE3
SmVAL-6	PF00188	Cysteine-rich secretory protein family	CRISP/PR-1/venom group 5 allergen family	G4V6N1
SmTropomysosin II	PF00261	Tropomyosin	Tropomyosin	G4VN65
SmLipocalin	PF00061	Lipocalin/cytosolic fatty-acid binding protein family	Lipocalin	G4M131
SmFurin-1	PF00082	Subtilase family	Subtilisin-like serine protease	G4VJB1

**Table 2:** **Identification of allergen-like proteins in abundant proteins from *S. mansoni* as identified in proteomic studies by Curwen et al. (21)**.

Protein name	Pfam domain	Pfam name	Allergen family name (Allfam)	Uniprot accession
Sm Calponin	PF00307	Calponin homology (CH) domain	EB1 family	G4M0V2
Sm Calreticulin	PF00262	Calreticulin family	Calreticulin family	Q06814
Sm HSP20 (p40)	PF00011	HSP20/alpha crystallin family	HSP20 heat shock protein	P12812
Sm Phosphoglycerate kinase	PF00162	Phosphoglycerate kinase	Phosphoglycerate kinase	P41759
Sm Superoxide dismutase	PF00080	Copper/zinc superoxide dismutase (SODC)	Cu/Zn Superoxide dismutase	Q01137
Sm GAPDH	PF02800	Glyceraldehyde 3-phosphate dehydrogenase, C-terminal domain	Glyceraldehyde 3-phosphate dehydrogenase	P20287
Sm HSP86	PF00183	Hsp90 Protein	Heat shock protein Hsp90	Q26582
Sm Aldehypde dehydrogenase	PF00171	Aldehyde dehydrogenase family	Aldehyde dehydrogenase	G4LWI3
Sm Serpin-like protein	PF00079	Serpin (serine protease inhibitor)	Serpin serine protease inhibitor	G4LZN6
Sm Triosephosphate Isomerase	PF00121	Triosephosphate Isomerase	Triosephosphate isomerase	G4V6B9
Sm Cyclophilin	PF00160	Cyclophilin type peptidyl-prolyl cis-trans isomerase/CLD	Cyclophilin	Q26565
Sm HSP70	PF00012	Hsp70 protein	Heat shock protein Hsp 70	G4V8L4
Sm GST26	PF00043	Glutathione S-transferase, C-terminal domain	Glutathione S-transferase	G4LXF8
Sm GST28	PF00043	Glutathione S-transferase, C-terminal domain	Glutathione S-transferase	P09792
Sm Thioredoxin	PF00085	Thioredoxin	Thioredoxin	Q8T9N5
Sm Taurocyamine kinase (ATP:GK)	PF00217	ATP:guanido phosphotransferase, C-terminal catalytic domain	ATP:guanido phosphotransferase	P16641
Sm Enolase	PF00113	Enolase, C-terminal TIM barrel domain	Enolase	G4VQ58
Sm FaBP/Sm Lipocalin	PF00061	Lipocalin/cytosolic fatty-acid binding protein family	Lipocalin	P29498/G4M131
Sm Tropomyosin-1	PF00261	Tropomyosin	Tropomyosin	P42637
Sm Myosin LC	PF13499	EF-hand domain pair	EF-hand domain	Q9Y1U7
Sm Calpain	PF00036	EF-hand	EF-hand domain	P27730
Sm TAL3	PF00036	EF-hand	EF-hand domain	P91804
Sm TAL2	PF00036	EF-hand	EF-hand domain	P32070
Sm E16	PF00036	EF-hand	EF-hand domain	Q07167
Sm 14-3-3 homolog 1	PF00244	14-3-3 protein	–	Q26540
Sm 14-3-3 ε	PF00244	14-3-3 protein	–	Q9U491
Sm Actin	PF00022	Actin	–	P53470
Sm AK	PF00406	Adenylate Kinase	–	P25824
Sm Dynein Light Chain	PF01221	Dynein light chain type 1	–	Q94758
Sm Ubiquitin	PF00240	Ubiquitin family	–	Q6RYS0
Sm Aldolase	PF00274	Fructose-bisphosphate aldolase class-I	–	P53442
Sm Elong F 1	PF00009	Elongation factor Tu GTP binding domain	–	Q94747

*For proteins with functional similarity to known allergens the Allfam allergen family name is given*.

From these two approaches, nine predicted parallergens were selected for study, SmProfilin, SmVAL-6, SmLipocalin, SmThioredoxin, SmCyclophilin, SmTPI, SmSOD, SmPGK, and SmHSP20. Additionally, 5 *S. mansoni* proteins were selected that were abundant within the parasite, but were not predicted to be parallergens, including SmAldolase, SmUbiquitin, SmDLC, SmAK, and Sm14-3-3. Finally, known IgE target proteins, SmTAL1, SmTAL2, and SmTropomyosin II produced for other studies were used as positive controls (17, 24).

### Cloning and expression of antigens in *E. coli*

**Figure 1:**
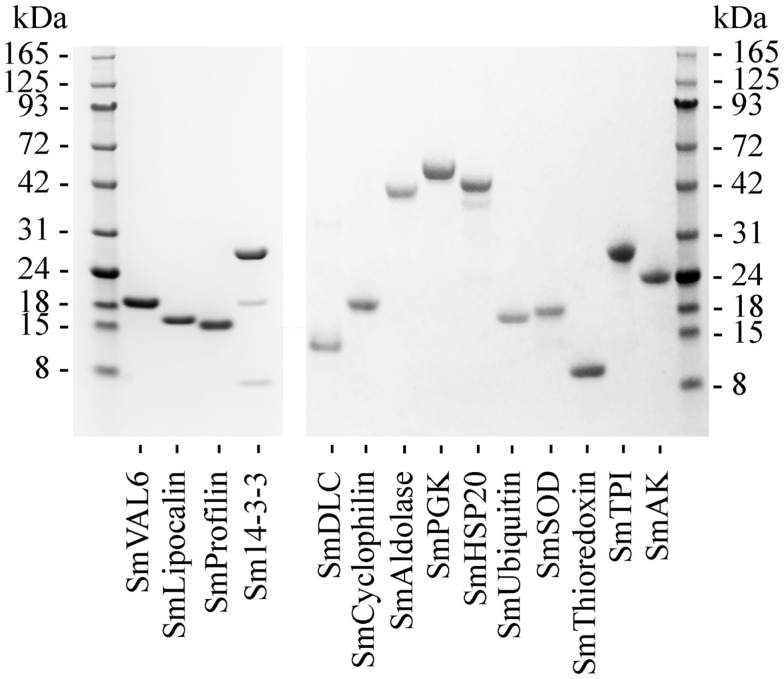
**SDS-PAGE of recombinant antigens following expression and purification**. Proteins were run on a 4–12% gradient Bis-Tris gel and stained with Coomassie Blue.

Recombinant forms of the antigens selected for cloning were produced in *E. coli*. In the case of SmVAL-6 only the soluble invariant N-terminal segment of the protein (amino acids 1–145) containing the CAP domain was expressed. For each protein, coding sequences were cloned from parasite cDNA by PCR. These were inserted into either the pGEX-KG or pET-30a vector and sequenced. Following expression and extensive purification molecular weights of the recombinant proteins were estimated by SDS-PAGE (Figure 1); SmVAL-6, 19 kDa; SmLipocalin, 15.5 kDa; SmProfilin, 15 kDa; Sm14-3-3, 26 kDa; SmDLC, 12 kDa; SmCyclophilin, 20 kDa; SmAldolase, 41 kDa; SmPGK, 45 kDa; SmHSP20, 42 kDa; SmUbiquitin, 16 kDa; SmSOD 17 kDa; SmThioredoxin 10 kDa; SmTPI 27 kDa; and SmAK 24 kDa. In the Sm14-3-3 sample, proteins observed at approximately 18 and 8 kDa represent the C and N-terminal (respectively) degradation products of full-length Sm14-3-3 as confirmed by MALDI fingerprinting (data not shown). Assay by ELISA confirmed that all recombinant proteins were free from significant contamination from the GST-fusion partner and bacterial antigens (data not shown).

### IgE responses are prevalent against predicted allergen-like molecules

In order to determine the immunogenicity of the antigens, serological responses were studied in detail. For each antigen, three antibody isotypes, IgE, IgG4, and IgG1, were measured in plasma from an infected cohort living in a schistosomiasis mansoni endemic area of Uganda. Previous studies have indicated that some antigens may be sequestered within the adult worms and not exposed to the immune system (24). Therefore antibody levels were studied 5 weeks post-treatment with PZQ so as to observe immune responses that may only be generated following worm damage or death. It is recognized that the use of a single dilution of sera per antibody isotype is a limitation of this study but it was a necessity given the number of individuals and sera isotypes assayed.

**Figure 2:**
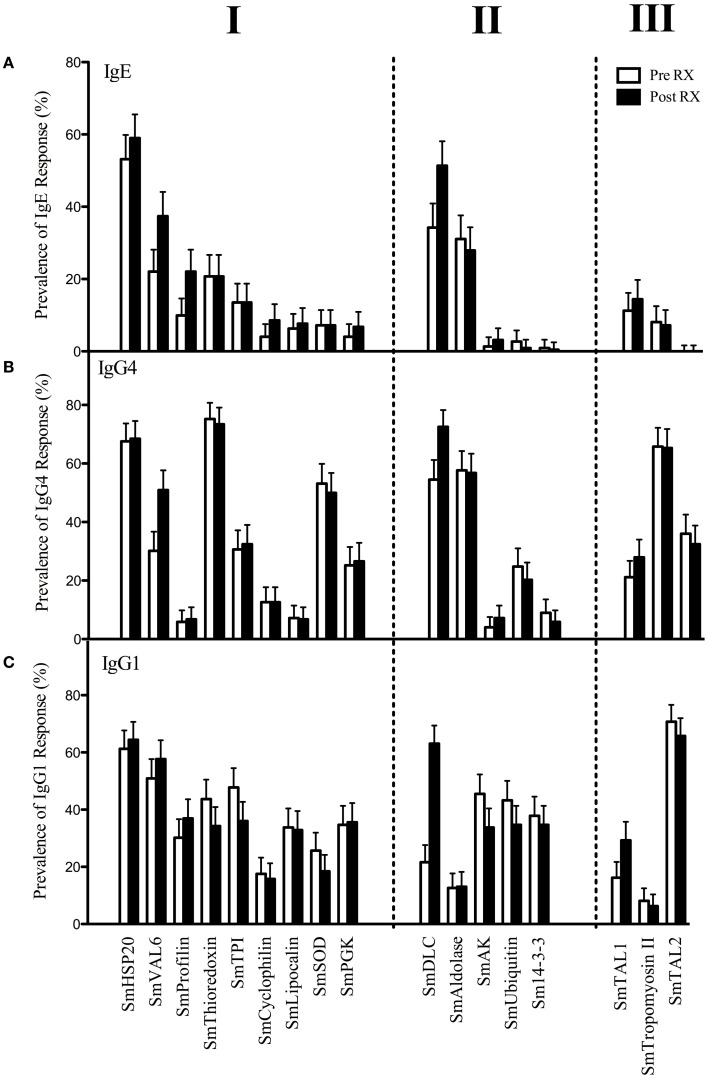
**Prevalence of IgE (A), IgG4 (B), and IgG1 (C) responses (with 95% binomial CIs), both pre- and post-treatment with PZQ to recombinant antigens within the population (*n* = 222)**. Prevalences/proportions of responders were calculated following the application of assay sensitivity cut-offs (mean + 3σ) determined using sera from 26 normal European controls. Proteins were grouped into predicted IgE-binding antigens (I), antigens without known allergenic domains (II), and confirmed IgE-binding antigens (III).

All nine of the predicted parallergens including SmHSP20, SmVAL-6, SmProfilin, SmThioredoxin, SmTPI, SmCyclophilin, SmLipocalin, SmSOD, and SmPGK were found to be the target of an IgE response both pre- and post-treatment (RX) with PZQ within varying portions of the population (Figure 2A, I). The proportions ranged from 59% post-RX for SmHSP20 to 4.1% pre-RX for SmPGK. For these antigens, the magnitudes of IgE responses ranged from 0.15 ng/ml for SmPGK pre-RX and 159.4 ng/ml for SmVAL-6 post-RX (Table 3). IgE responses were also seen against the positive control parallergens SmTAL1 and SmTropomyosin II (Figure 2A, III). Significant boosts in the magnitude of IgE responses following treatment occurred for SmProfilin, SmVAL-6, SmHSP20, and SmDLC (Table 3).

**Table 3:** **Magnitudes of antibody responses against recombinant *S. mansoni* antigens in a population of 222 infected individuals as geometric means (Ab magnitude + 0.01) of IgG1, IgG4, and IgE for sub populations of individuals with responses to *S. mansoni* antigens either pre- or post-treatment with PZQ**.

Antigen		IgE (ng/ml)	IgG4 (μg/ml)	IgG1 (μg/ml)
SmTAL1	Pre-RX	3.96 (0.79, 19.89)	0.09 (0.06, 0.14)	33.4 (23.1, 48.2)
	Post-RX	17.7 (4.23, 73.87)	0.40 (0.23, 0.70)	50.7 (35.4, 72.6)
	Boost	346.3% (*n* = 42)	**347.9% (*n* = 76)**	**52.1% (*n* = 72)**
SmTAL2	Pre-RX	0 (0, 0)	0.20 (0.13, 0.30)	20.2 (15.8, 25.7)
	Post-RX	0 (0, 0)	0.31 (0.18, 0.54)	15.5 (11.3, 21.3)
	Boost	0% (*n* = 0)	**59.8% (*n* = 96)**	−23.2% (*n* = 169)
SmProfilin	Pre-RX	0.19 (0.07, 0.54)	0.35 (0.10, 1.24)	71.9 (55.0, 94.0)
	Post-RX	6.60 (2.65, 16.5)	1.09 (0.32, 3.78)	82.8 (60.9, 112.6)
	Boost	**3345.0% (*n* = 64)**	207.9% (*n* = 19)	15.1% (*n* = 96)
SmVAL-6	Pre-RX	19.5 (8.75, 43.6)	0.44 (0.24, 0.83)	8.5 (5.4, 13.4)
	Post-RX	159.4 (105.5, 240.8)	11.0 (7.00, 15.2)	25.1 (15.6, 40.2)
	Boost	**715.7% (*n* = 88)**	**2386.6% (*n* = 117)**	**194% (*n* = 154)**
SmTropomyosin II	Pre-RX	114.6 (88.1, 149.0)	8.11 (6.49, 10.1)	151.1 (115.6, 197.6)
	Post-RX	93.5 (63.3, 137.9)	8.68 (6.95, 10.8)	117.4 (92.2, 149.7)
	Boost	−18.4% (*n* = 24)	7.0% (*n* = 160)	−22.3% (*n* = 20)
SmLipocalin	Pre-RX	3.68 (0.45, 30.1)	0.05 (0.03, 0.10)	8.1 (4.4, 14.7)
	Post-RX	12.0 (1.84, 77.6)	0.03 (0.02, 0.06)	6.8 (3.6, 12.3)
	Boost	224.9% (*n* = 23)	−34.0% (*n* = 20)	−16.1% (*n* = 86)
SmThioredoxin	Pre-RX	16.5 (5.34, 50.8)	9.60 (6.50, 14.2)	54.7 (28.7, 104.1)
	Post-RX	16.7 (5.41, 51.6)	9.37 (6.23, 14.1)	8.2 (3.4, 19.7)
	Boost	1.4% (*n* = 62)	−2.4% (*n* = 181)	**−85.0% (*n* = 112)**
SmCyclophilin	Pre-RX	0.23 (0.04, 1.35)	0.42 (0.22, 0.82)	80.2 (68.8, 93.6)
	Post-RX	4.75 (1.09, 20.7)	0.46 (0.26, 0.82)	70.8 (61.8, 81.0)
	Boost	1979.6% (*n* = 25)	9.6 (*n* = 36)	**−11.8% (*n* = 44)**
SmTPI	Pre-RX	1.60 (0.57, 4.47)	0.44 (0.27, 0.71)	12 (8.0, 18.4)
	Post-RX	1.49 (0.54, 4.13)	0.64 (0.40, 1.04)	1.9 (1.0, 3.8)
	Boost	−6.9% (*n* = 41)	**46.8% (*n* = 88)**	**−84.0% (112)**
SmSOD	Pre-RX	21.7 (11.7, 40.3)	0.30 (0.22, 0.40)	120.4 (103.5, 134.0)
	Post-RX	18.6 (12.3, 28.1)	0.23 (0.17, 0.31)	97.7 (81.9, 116.6)
	Boost	−14.4% (*n* = 24)	**−23.0% (*n* = 144)**	**−18.9% (*n* = 75)**
SmPGK	Pre-RX	0.15 (0.04, 0.61)	0.32 (0.12, 0.83)	1.4 (0.8, 2.8)
	Post-RX	0.78 (0.26, 2.36)	0.75 (0.26, 2.12)	1.7 (1.0, 3.2)
	Boost	434.2% (*n* = 18)	**132.6% (*n* = 66)**	20.9% (*n* = 97)
SmHSP20	Pre-RX	8.24 (4.54, 14.9)	297.5 (185.6, 477.0)	40.0 (22.9, 70.0)
	Post-RX	22.7 (13.7, 37.7)	319.6 (207.7, 491.7)	71.8 (44.9, 114.9)
	Boost	**176.0% (*n* = 147)**	**7.4% (*n* = 160)**	79.5% (*n* = 157)
SmAldolase	Pre-RX	3.64 (1.66, 7.98)	0.30 (0.22, 0.39)	64.9 (48.2, 87.2)
	Post-RX	2.19 (0.95, 5.06)	0.29 (0.22, 0.39)	63.9 (49.1, 83.2)
	Boost	−39.8% (*n* = 98)	−1.7% (*n* = 148)	−1.7% (*n* = 38)
SmDLC	Pre-RX	32.4 (15.8, 66.3)	0.14 (0.09, 0.21)	0.2 (0.1, 0.3)
	Post-RX	209.4 (144.1, 304.4)	0.49 (0.33, 0.73)	120.1 (80.2, 179.9)
	Boost	**546.7% (*n* = 124)**	**248.0% (*n* = 173)**	**70891% (*n* = 147)**
SmUbiquitin	Pre-RX	3.78 (0.23, 61.3)	1.35 (0.71, 2.56)	13.4 (8.5, 21.1)
	Post-RX	0.07 (0.00, 1.82)	0.59 (0.28, 1.25)	3.1 (1.6, 6.3)
	Boost	−98.0% (*n* = 7)	−56.0% (*n* = 69)	**−76.6% (*n* = 105)**
SmAK	Pre-RX	0.13 (0.01, 2.61)	0.08 (0.02, 0.28)	10.6 (6.5, 17.1)
	Post-RX	2.93 (0.22, 39.1)	0.22 (0.10, 0.49)	2.0 (1.0, 4.0)
	Boost	2118.1% (*n* = 9)	172.3% (*n* = 20)	**−81.4% (*n* = 96)**
Sm14-3-3	Pre-RX	2.33 (0.00, 309075)	0.84 (0.36, 1.94)	10.3 (5.9, 18.1)
	Post-RX	0.10 (0.00, 2569)	0.21 (0.06, 0.72)	6.1 (3.1, 11.9)
	Boost	−95.5% (*n* = 3)	**−75.2% (*n* = 21)**	**−41.3% (*n* = 96)**

*Changes in Ab magnitudes were calculated as described in the Materials and Methods. Significant differences in the levels of antibodies pre- and post-RX (Wilcoxon matched-pairs signed-ranks test *p* < 0.05) are highlighted in bold*.

**n*, Number of responders in each group*.

Of the five antigens that did not contain known allergenic domains, three of the antigens, SmAK, SmUbiquitin, and Sm14-3-3 were found to have low prevalences of IgE responders; 1.3/3.1%, 2.7/0.9%, and 0.9/0.45% pre-/post-RX, respectively (Figure 2A, II). Conversely SmDLC and SmAldolase had a relatively large proportion of specific IgE responders; 34.2/51.3% and 31.1/27.9% pre/post-RX, respectively. As shown in previous endemic study populations that did not include young children (<5 years), IgE responses to SmTAL2 were absent despite its similarity to allergens from the EF-hand family (15, 24).

### IgG4 responses are prevalent against allergen-like IgE-binding antigens

**Figure 3:**
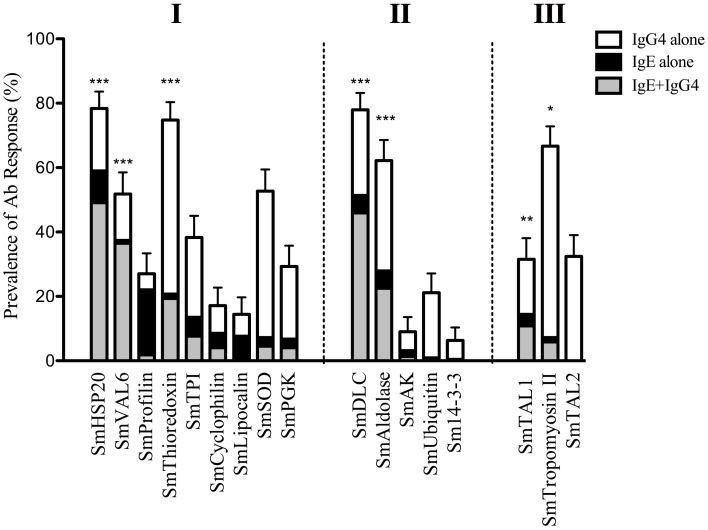
**Proportions of individuals responding with IgE alone, IgG4 alone, or IgG4 and IgE together post-treatment with PZQ**. Prevalences/proportions of responders were calculated following the application of assay sensitivity cut-offs (mean + 3σ) determined using sera from 26 normal European controls. Two-sample test of proportions was used to compare the prevalence of individuals responding with IgE alone vs. those responding with IgE and IgG4 together (*p < 0.05, **p < 0.01, ***p < 0.001). Proteins were grouped into predicted IgE-binding antigens (I), antigens without known allergenic domains (II), and confirmed IgE-binding antigens (III).

It has been suggested that production of IgG4 against *S. mansoni* allergen-like proteins can be induced as part of the regulatory response to IgE-mediated inflammation and should therefore be considered as part of an allergy-like response (24). In this study, IgG4 responses were observed against all of the IgE-binding antigens studied both pre- and post-RX with PZQ (Figure 2B) with the most prevalent against SmThioredoxin (75.2% pre-RX) and the least against SmProfilin (5.9% pre-RX). Furthermore for the parallergens SmHSP20, SmDLC, SmThioredoxin, SmTropomyosin II, SmAldolase, SmVAL-6, and SmTAL1 the majority of individuals that produced an IgE response also produced an IgG4 response against the same antigen with a significantly greater proportion of individuals producing both IgE and IgG4 responses than an IgE response alone (Figure 3). For SmAK and Sm14-3-3 to which low prevalence IgE responses were observed, IgG4 responses were also of low prevalence. Of note was SmTAL2, which despite the absence of IgE responses was the target of specific IgG4 responses in 35 and 32% of the population pre-/post-RX, respectively. Similarly in the case of SmUbiquitin, despite a low proportion of IgE responders in the population, prevalent IgG4 responses occurred.

### IgG1 responses are prevalent against recombinant *S. mansoni* proteins

All of the antigens, both allergen- and non-allergen-like, were found to elicit IgG1 responses in human infections both pre- and post-treatment with PZQ. IgG1 responses were found to be present against all of the recombinant antigens ranging in prevalence from 6.3% post-RX for SmTropomyosin II to 70.7% pre-RX for SmTAL2 (Figure 2C). Magnitudes of IgG1 response amongst responders were between 0.2 μg/ml pre-RX for SmDLC and 151.1 μg/ml pre-RX for SmTropomyosin II (Table 3). Significant differences in the magnitudes of IgG1 responses following treatment with PZQ were seen for SmTAL1, SmVAL-6, SmCyclophilin, SmThioredoxin, SmTPI, SmSOD, SmDLC, SmUbiquitin, SmAK, and Sm14-3-3 (Table 3).

### Parallergens and allergens have close conformational similarity

**Figure 4:**
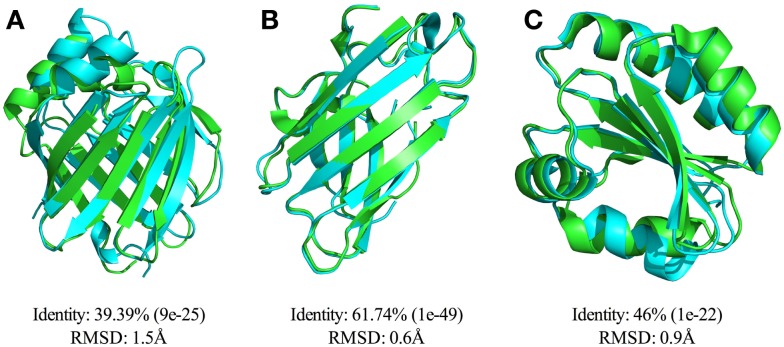
**Comparison of the structures of SmLipocalin, SmThioredoxin, SmSOD with their respective IgE-binding counterparts identified from Allergome**. Parasite allergen-like molecules were used to identify similar allergens by BLAST search of the Allergome database. Identities of the matched allergens with the corresponding *E*-values are shown below each matched pair. Structures were superimposed using LSQMAN and the quality of the alignment assessed by root mean squared deviation (RMSD). **(A)** Alignment of SmLipocalin (1VYF, cyan) and Der f 13 (2A0A, green). **(B)** Alignment of SmSOD (1TO4, cyan) and Tomato SOD (3HOG, green). **(C)** Alignment of SmThioredoxin (2XBI, cyan) and White Shrimp Thioredoxin (3ZZX, green).

Confirmed *S. mansoni* parallergens with previously elucidated 3D structures, SmLipocalin (1VYF), SmSOD (1TO4), and SmThioredoxin (2XBI), were compared for gross structural similarity to allergens with known 3D structure including *Dermatophagoides farinae* Der f 13 (2A0A), *Solanum lycopersicum* Superoxide dismutase (3HOG), and *Litopenaeus vannamei* Thioredoxin (3ZZX), respectively. All three proteins were found to have overall structural similarity with their counterpart allergens with RMSD scores of 0.9 Å, 0.6 Å, and 1.5 Å for SmThioredoxin/LvThioredoxin, SmSOD/SlSOD, and SmLipocalin/Der f 13, respectively (Figure 4).

### Antigens are differentially transcribed in *S. mansoni* adult and egg life cycle stages

**Figure 5:**
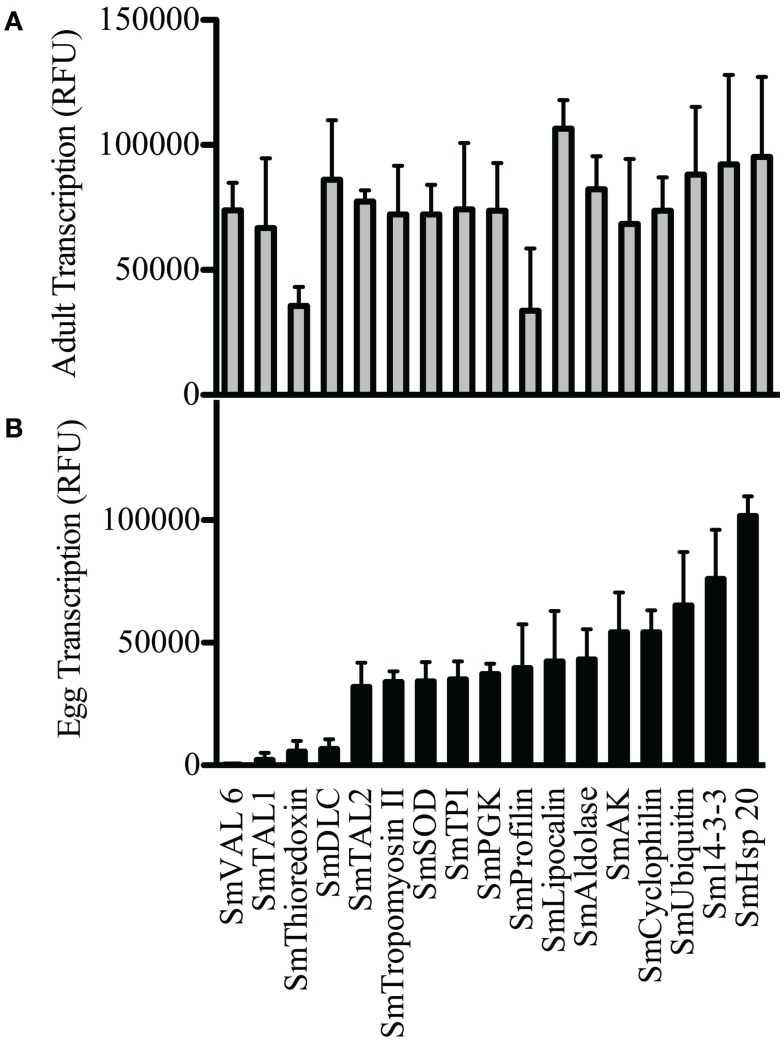
**Differential transcription of antigens in *S. mansoni* adult worms (A) and eggs (B) as determined by previously published microarray studies (35)**. Levels of antigen transcription were expressed as relative fluorescent units (RFU).

Previous studies have suggested that differing levels of *S. mansoni* antigen exposure may influence the prevalence and magnitude of antigen-specific antibody responses (15, 24). Available microarray data (35) show that all of the antigens in this study were transcribed in adult worms (Figure 5A), and whilst transcription was not equal across all antigens (SmThioredoxin and SmProfilin were found to be significantly lower in expression than all of the antigens) there were no antigens for which transcription in adult worms was very low or absent. Transcription of antigens in eggs (Figure 5B) was more selective with some antigens showing significantly lower expression than others (SmVAL-6, SmTAL1, SmThioredoxin, SmDLC vs. all other antigens, *p* < 0.001) and some significantly higher than others (SmHSP20 vs. all other antigens *p* < 0.001). SmVAL-6 and SmDLC, both transcribed at low-levels in eggs (0.5 and 7.7% of adult levels, respectively) showed marked boosts in antibody levels following treatment, whilst those more strongly expressed in egg, such as SmSOD, SmUbiquitin, and Sm14-3-3 (between 47.5 and 82.5% of adult transcription levels) all showed marked decreases in antibody levels post-treatment (Table 3).

### Antigen-specific levels of IgE are negatively correlated with antigen expression in eggs

**Figure 6:**
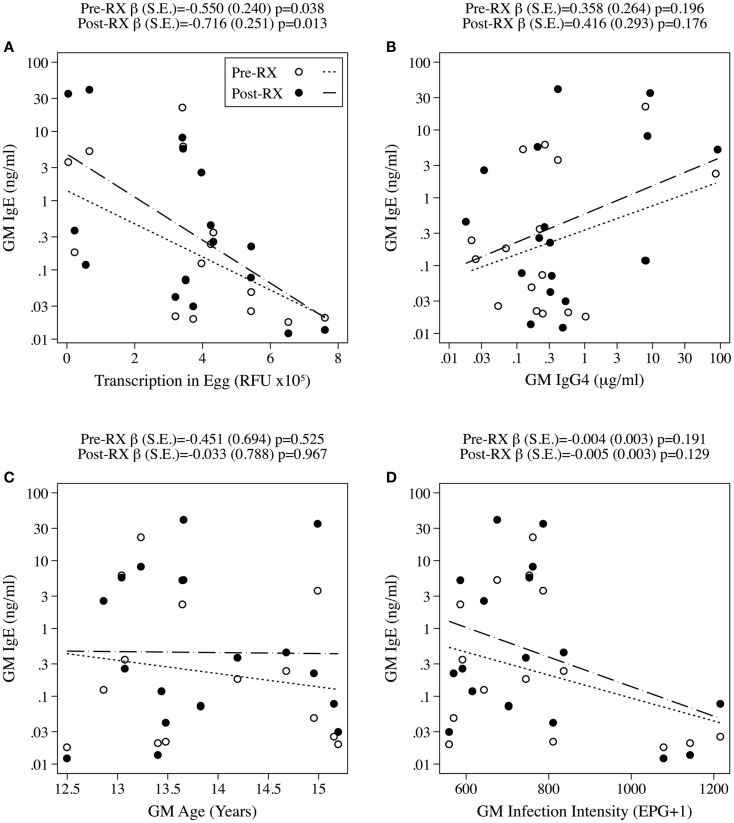
Relationships between antigen-specific natural log normalized geometric mean (GM) IgE levels and transcription of antigen in egg (A)*, natural log normalized GM IgG4 levels (B), GM age (C), or GM infection intensity (D) in individuals that respond to either IgG4 or IgE both pre- (open circles, short dashes) and post-treatment (filled circles, long dashes) with PZQ to a given antigen (β, SE, and *p* value calculated by linear regression). *SmHSP20 was excluded from this plot as it exerted undue influence on the linear regression model (Cook’s distance > 1).

In order to determine if constant exposure of antigens from the death of short-lived eggs in host tissues influenced levels of IgE, we compared the level of antigen expression in eggs with antigen-specific IgE levels in infected individuals both pre- and post-treatment with PZQ. As previous studies have demonstrated that the age of infected individuals, their level of IgG4 antibodies and intensity of infection (as measured by number of eggs in feces) may confound the levels of IgE antibodies, these variables were included in univariate regression models explaining variation in IgE magnitude (Figure 6) (17, 29, 36–38). Regression diagnostics indicated that SmHSP20 exerted undue influence over the effect of egg transcription on IgE levels (Cook’s distance > 1) and was therefore excluded from this model. Upon the exclusion of SmHSP20 this analysis established that antigen-specific IgE magnitude prior to treatment was significantly negatively correlated with transcription levels for each antigen in eggs (β = −0.550, *r*^2^ = 0.2739, *p* = 0.038) an association that strengthened post-treatment with PZQ (β = −0.716, *r*^2^ = 0.3666, *p* = 0.013) as shown in Figure 6A. None of the other confounding variables, including geometric mean (GM) age, GM infection intensity, or GM IgG4 levels were found to significantly correlate with IgE levels.

## Discussion

Allergy and atopic spectrum diseases have been extensively studied over a number of decades providing a detailed description of many of the protein allergens that are associated with IgE-mediated allergic immune reactions (1, 3). We have suggested that the immune responses associated with allergy are the by-product of the highly evolved immune response to metazoan parasites, and that allergens and metazoan IgE-binding proteins share similar structural features, which define the immune responses produced against them (5). To this end, we hypothesized that known protein allergen structures could be used to predict IgE-binding proteins from the metazoan parasite *S. mansoni*.

We used a bioinformatic approach to search for *S. mansoni* parallergen examples of abundant common allergens and to predict parallergens in proteins abundant in *S. mansoni*. Nine potential IgE-binding antigens were cloned in *E. coli* alongside, three known IgE targeted *S. mansoni* antigens, and five other abundant *S. mansoni* antigens that were predicted not to bind IgE due to the lack of known similar proteins in allergy. We then tested if these antigens were targets for IgE, IgG4, and IgG1 in individuals infected with the parasite.

IgE responses were prevalent against all of the predicted parallergens, and against the known IgE target control antigens SmTAL1 and SmTropomyosin II. This study defines a novel set of antigens that are targeted by IgE in plasma from individuals with schistosomiasis mansoni, including SmProfilin, SmHSP20, SmPGK, SmSOD, SmCyclophilin, and SmThioredoxin. Several of these antigens are also the first examples of parallergens with similarity to known environmental allergens from the Profilin, HSP20 heat shock protein, Cu/Zn Superoxide dismutase, and Thioredoxin allergen families, respectively. SmVAL-6 is a novel human IgE-binding parallergen in *S. mansoni* and along with previously identified hookworm Na-ASP-2 and SmVAL-4 parallergens is a further example of a parallergen with structural similarity to allergens from the CAP family (18, 39). IgE-binding to Cyclophilin from *Echinococcus granulosus* has also been previously reported in infected humans (40). IgE responses against SmTAL1 and SmTropomyosin II have been previously reported in *S. mansoni* endemic areas of Uganda, whilst responses against SmTPI and SmLipocalin were described in a recent study on infected indviduals from Brazil (15, 17, 19). Despite being a predicted IgE targeted antigen, SmTAL2 was not targeted by IgE. Other studies on human antibody responses against SmTAL2 in schistosomiasis mansoni endemic areas have shown that SmTAL2 may be targeted by IgE but only in early infection, which typically occurs in children under the age of 5, an age group not present in our study (24, 29).

Of the five *S. mansoni* antigens without allergen family counterparts, there was no strong evidence of IgE targeting of SmAK, SmUbiquitin, or Sm14-3-3; IgE responses were essentially absent from the cohort, being of both low prevalence and magnitude. SmDLC and SmAldolase however, were both targets of prevalent IgE responses. Currently, the Allfam dataset used to identify IgE-binding proteins in this study only identifies class II aldolases (Pfam Acc. PF01116) as allergens, whilst SmAldolase is a class-I aldolase (Pfam Acc. PF00274) with distinctive structure associated with a separate Pfam domain (41). However, recent proteomic work has shown that a class-I Aldolase from maize (*Zea mays*) is targeted by IgE in maize allergic individuals, confirming SmAldolase as an allergen homolog (42, 43). Previous work on SmDLC had indicated that it was not the target of an IgE response (15), however that study used antigens that had been prepared under denaturing conditions, which may have affected their antigenicity. The evidence here that SmDLC is part of a novel class of IgE-binding proteins in *S. mansoni*, despite their absence from the allergen databases, may point to an important difference between allergens and some parallergens. In order to interact with the sufferer, allergens require the stability to survive the environment and cross mucosal barriers (44), whilst parallergens can be delivered directly into the blood and tissues by the parasite. Hence some classes of parasite IgE-binding proteins, such as DLC, may be absent from the allergy datasets as they are simply not seen by the immune systems of atopic individuals. Additionally, as illustrated for SmAldolase, the Allfam dataset, whilst comprehensive, is far from complete and it may be as more detailed proteomic studies of known allergen sources are completed further novel IgE-binding families are identified. For example, in a 2014 proteomic study on maize allergens, 6 of the 11 novel allergens found were not previously described in other allergenic sources and thus far are not present as Allfam families (42).

In the case of antibody responses to allergens in patients undergoing SIT and parasite IgE-binding proteins in chronic infections, IgG4 is thought to be induced as part of a protective anti-inflammatory regulatory response to IgE-mediated inflammation (reviewed (45) and (23), respectively). IgG4 responses were observed against all of the allergen-like antigens in this study in varying amounts. For SmHSP20, SmDLC, SmThioredoxin, SmTropomyosin II, SmAldolase, SmVAL-6, and SmTAL1, the majority of individuals that produced an IgE response against a given antigen also produced an IgG4 response against the same antigen providing further evidence for the role of IgG4 in a protective anti-inflammatory role in humans chronically infected with *S. mansoni*. Prevalent IgG4 responses were also observed against molecules with low prevalence IgE responses including SmTAL2 and SmUbiquitin. As discussed previously, it is thought that for antigens expressed strongly in eggs, such as SmTAL2, IgE antibodies are seen only during early infection (children <5 years) with IgE responses down regulated and replaced with IgG4 antibody responses in older individuals (24, 29). It is possible that this may also occur for SmUbiquitin, which is also expressed strongly in eggs, and that its absence from databases of known allergens is due to a similar process in which exposure to Ubiquitin, which is highly conserved in eukaryotes and abundant in other allergen sources such as birch pollen (46, 47), in atopic individuals results in down regulated IgE and up regulated IgG4.

Importantly, all of antigens tested were immunogenic, with differing proportions of infected individuals within the population producing an IgG1 response against each of the antigens. For several of these (SmPGK, SmSOD, SmLipocalin, SmTAL1, SmTAL2, SmTropomyosin II, Sm14-3-3, SmTPI, and SmDLC), responsiveness has been shown previously in infected humans and mice (15, 17, 48–52). However, novel human IgG1 responses were described to SmAK, SmUbiquitin, SmAldolase, SmHSP20, SmCyclophilin, SmThioredoxin, SmVAL-6, and SmProfilin. Crucially for Sm14-3-3, SmUbiquitin, and SmAK, to which IgE responses were low, a prevalent IgG1 response was found, confirming that the low-level of allergy-like responses was not due to poor immunogenicity.

Alignments of known parallergen and allergen structures including examples of Superoxide dismutase, Thioredoxin, and Lipocalin allergen families demonstrated that, despite relatively low sequence identity between matched parallergens and allergens, there was high gross structural similarity. This observation lends further weight to our hypothesis, which suggests that the structural similarity between allergens and metazoan parasite IgE-binding molecules defines the restriction of allergen families to a small proportion of structural domains. We predict that more extensive bioinformatic, crystallographic, and serological studies of whole parasite proteomes and the five remaining allergen super families (Prolamins, Subtilisin-like serine proteases, Bet v1-like proteins, Cupins, and Expansins) may reveal further parallergens in support of this hypothesis.

Studies on the SmTAL family of antigens suggest that sequestration of antigens may influence their overall levels of exposure to the immune system and consequently antibody levels (24). In schistosomiasis, the death of eggs trapped in tissue has been suggested to result in constant exposure of allergen-like molecules to the immune system, whilst pharmacological damage of adult worms results in exposure to sequestered antigens upon treatment with PZQ (15, 24). Consistent with this IgE, IgG4 and IgG1 antibody levels to both SmVAL-6 and SmDLC, antigens that are expressed in adult worms but not eggs, boosted significantly following treatment with PZQ. Conversely antigens expressed in both eggs and adults, including SmSOD, Sm14-3-3, and SmUbiquitin had significant decreases in specific antibody levels 5 weeks post-treatment with PZQ, which may be the result of decreased exposure to these antigens as eggs are no longer being produced. Previous investigations of serological responses in SIT have indicated that prolonged exposure to allergens may result in a decrease in serum IgE levels (26, 53). We found that for the majority of antigens in the study the relative level of expression of antigens in egg was negatively correlated with levels of IgE to that antigen. However, levels of SmHSP20 specific IgE were high despite being the molecule being strongly expressed in eggs. Intriguingly, the levels of IgG4 to SmHSP20 were between 30 and 35 times greater than next highest antigen-specific IgG4 levels (SmThioredoxin). Therefore we suggest that, for the majority of antigens, constant exposure to antigens due to the death of eggs results in a down-regulation of IgE against those antigens with the effective dose of antigen received (as indicated by transcription level in eggs) governing the level of IgE suppression. The data for SmHSP20 suggest that there may also be another mechanism for regulating deleterious IgE responses in which large quantities of IgG4 are produced in the absence of IgE down-regulation, the reasons for which require further investigation. The parallels between SIT and the natural immune response to chronic *S. mansoni* infection described here and elsewhere highlight the similarities between the immune responses to metazoan parasites and allergens and provide further evidence for the evolutionary origins of allergy-like responses in the immune reactions against metazoan parasites.

Our data, supporting the hypothesis that molecular structures associated with metazoan parasites determine the allergen landscape, have broad implications for the evolution and function of the IgE response consistent with its role of protection against these organisms. Endo- and ecto-macroparasites would have placed strong evolutionary pressure on the immune system, and specialized molecular mechanisms are likely to have developed to rapidly expand IgE responsiveness during parasite challenge and regulate harmful IgE-mediated immune responses. Whether operating through an antigen-specific mechanism, or invoking a new set of pathogen receptors, this hypothesis provides a novel, testable, explanation of allergy and allergens (54). In this setting, a more detailed analysis of functionally diverse *S. mansoni* antigens, guided by a structural bioinformatics pipeline based on known allergen structures, may provide a clearer picture as to the complex relationship between IgE production, regulatory IgG4 responses, and protective responses to the parasite and help elucidate the mechanisms of IgE targeting in both parasitic disease and allergy.

## Conflict of Interest Statement

The authors declare that the research was conducted in the absence of any commercial or financial relationships that could be construed as a potential conflict of interest. The Review Editor Adam Cunningham declares that, despite having collaborated on a publication in the last 2 years with author Rick M. Maizels, the review process was handled objectively.

## Supplementary Material

The Supplementary Material for this article can be found online at http://www.frontiersin.org/Journal/10.3389/fimmu.2015.00026/abstract

## Footnotes

^1^www.meduniwien.ac.at/allergens/allfam/

^2^http://www.uniprot.org

^3^http://www.allergome.org/

^4^http://www.ebi.ac.uk/pdbe/

^5^http://xray.bmc.uu.se/usf/lsqman_man.html

^6^http://primer3plus.com
