# Supplementary material for: Known Allergen Structures Predict Schistosoma mansoni IgE-Binding Antigens in Human Infection
Source: Front Immunol. 2015 Feb 3;6:26. doi: 10.3389/fimmu.2015.00026 (PMC4315118; doi:10.3389/fimmu.2015.00026)
Supplement: Supplementary file 1 [file Table1.DOCX]

***Supplementary Material***

Known allergen structures predict *Schistosoma mansoni* IgE binding antigens in human infection

**Edward J Farnell^1^*, Nidhi Tyagi^2^, Stephanie Ryan^3^, Iain W Chalmers^4^, Angela Pinot de Moira^1^, Frances M Jones^1^, Jakub Wawrzyniak^1^, Colin M Fitzsimmons^1^, Edridah M Tukahebwa^5^, Nicholas Furnham^2,6^, Rick M Maizels^3^ and David W Dunne^1^**

^1.^Department of Pathology, University of Cambridge, UK

^2.^European Bioinformatics Institute, Cambridge, UK

^3.^Institute of Immunology and Infection Research, University of Edinburgh, UK

^4.^Institute of Biological, Environmental and Rural Sciences, Aberystwyth University, UK.

^5.^Vector Control Division, Ugandan Ministry of Health, Uganda

^6.^Department of Pathogen Molecular Biology, London School Hygiene and Tropical Medicine, UK

*** Correspondence:** Edward J Farnell, Department of Pathology, University of Cambridge, Tennis Court Road, Cambridge, CB2 1QE

ef242@cam.ac.uk

**Supplementary Table S1.** Primers, annealing temperatures, and restriction enzymes used for PCRs in the cloning and expression of *S. mansoni* antigens.

| Antigen | Primer | Tm (°C) | Restriction Enzymes |
| --- | --- | --- | --- |
| SmVAL6 | 5’-TCTAGAATGATTAATGAACGTTTTAATGAT-3’  5’-CTCGAGTGGTACATTCCATCCG-3’ | 66 | XhoI/XbaI |
| SmProfilin | 5’-TAGAATTCTAATGTCCGAAGAGTGGAAAAA-3’  5’-TACTCGAGATACATGGGCAATATTTATTGG-3’ | 68 | XbaI/XhoI |
| SmLipocalin | 5’-TATCTAGAGATGTCTAGTTTCTTGGGAAAG-3’  5’-ATCTCGAGTTAGGATAGTCGTTTATAATTGC-3’ | 61 | XbaI/XhoI |
| SmThioredoxin | 5’- TATCTAGAGATGTCTAAGCTGATTGAA -3’  5’- ATCTCGAGTTAAATAAATTTCTTGATCA -3’ | 60 | XbaI/XhoI |
| SmPGK | 5’- TATCTAGAGATGGTCGGCTGC -3’  5’- ATCTCGAGTTAATGAGCATCCG -3’ | 63 | XbaI/XhoI |
| SmSOD | 5’- TATCTAGAGATGAAAGCTGTTTGTGT -3’  5’- ATCTCGAGCTACTCAGCAGC -3’ | 61 | XbaI/XhoI |
| SmHSP20 | 5’- TATCTAGAGATGTCTGGTGGGA -3’  5’- ATCTCGAGCTAGTGAGTAATTGCA -3’ | 60 | XbaI/XhoI |
| SmCyclophilin | 5’- TATCTAGAGATGGCTGCGAAAGC -3’  5’- ATCTCGAGTTAACATTCTCCACA -3’ | 61 | XbaI/XhoI |
| SmAldolase | 5’- TATCTAGAGATGTCACGCTTCCA -3’  5’- ATCTCGAGTTAATAAGCGTGA -3’ | 58 | XbaI/XhoI |
| SmAK | 5’- TAGAATTCTAATGACTGATCAGAAGTTAGC -3’  5’- ATCTCGAGTTATTTCACACCGAAT -3’ | 61 | EcoRI/XhoI |
| SmUbiquitin | 5’- TATCTAGAGATGCAAATATTCGTGA -3’  5’- ATCTCGAGTCAACCTCCACG -3’ | 60 | XbaI/XhoI |
| Sm14-3-3 | 5’-TAGAATTCTAATGACTACGTCGTGG-3’  5’-ATCTCGAGTTAGCTGTCATTTTCA-3’ | 63 | EcoRI/XhoI |
