# Supplementary material for: Known Allergen Structures Predict Schistosoma mansoni IgE-Binding Antigens in Human Infection
Source: Front Immunol. 2015 Feb 3;6:26. doi: 10.3389/fimmu.2015.00026 (PMC4315118; doi:10.3389/fimmu.2015.00026)
Supplement: Supplementary file 2 [file Table2.DOCX]

***Supplementary Material***

Known allergen structures predict *Schistosoma mansoni* IgE binding antigens in human infection

**Edward J Farnell^1^*, Nidhi Tyagi^2^, Stephanie Ryan^3^, Iain W Chalmers^4^, Angela Pinot de Moira^1^, Frances M Jones^1^, Jakub Wawrzyniak^1^, Colin M Fitzsimmons^1^, Edridah M Tukahebwa^5^, Nicholas Furnham^2,6^, Rick M Maizels^3^ and David W Dunne^1^**

^1.^Department of Pathology, University of Cambridge, UK

^2.^European Bioinformatics Institute, Cambridge, UK

^3.^Institute of Immunology and Infection Research, University of Edinburgh, UK

^4.^Institute of Biological, Environmental and Rural Sciences, Aberystwyth University, UK.

^5.^Vector Control Division, Ugandan Ministry of Health, Uganda

^6.^Department of Pathogen Molecular Biology, London School Hygiene and Tropical Medicine, UK

*** Correspondence:** Edward J Farnell, Department of Pathology, University of Cambridge, Tennis Court Road, Cambridge, CB2 1QE

ef242@cam.ac.uk

**Supplementary Table S2.** Coating concentrations and conditions for antigens used in ELISA assays as described in materials and methods

| Antigen | Coating concentration  (μg/ml) | Coating conditions |
| --- | --- | --- |
| SmTAL1 | 18.0 | 4^0^C overnight |
| SmTAL2 | 18.0 | 4^0^C overnight |
| SmTPM2.8 | 2.5 | 37^0^C overnight  (dried down) |
| SmThioredoxin | 20.0 | 4^0^C overnight |
| SmCyclophilin | 6.4 | 37^0^C overnight  (dried down) |
| SmTPI | 29.1 | 4^0^C overnight |
| SmSOD | 3.2 | 37^0^C overnight  (dried down) |
| SmPGK | 11.6 | 4^0^C overnight |
| SmHSP20 | 5.0 | 4^0^C overnight |
| SmAldolase | 7.8 | 37^0^C overnight  (dried down) |
| SmUbiquitin | 3.44 | 37^0^C overnight  (dried down) |
| SmDLC | 3.75 | 4^0^C overnight |
| SmAK | 10.0 | 4^0^C overnight |
| SmVAL6 | 3.75 | 37^0^C overnight  (dried down) |
| SmProfilin | 8 | 37^0^C overnight  (dried down) |
| SmLipocalin | 10 | 37^0^C overnight  (dried down) |
| Sm14-3-3 | 20 | 4^0^C overnight |
