# Supplementary material for: Known Allergen Structures Predict Schistosoma mansoni IgE-Binding Antigens in Human Infection
Source: Front Immunol. 2015 Feb 3;6:26. doi: 10.3389/fimmu.2015.00026 (PMC4315118; doi:10.3389/fimmu.2015.00026)
Supplement: Supplementary file 3 [file Table3.DOCX]

***Supplementary Material***

Known allergen structures predict *Schistosoma mansoni* IgE binding antigens in human infection

**Edward J Farnell^1^*, Nidhi Tyagi^2^, Stephanie Ryan^3^, Iain W Chalmers^4^, Angela Pinot de Moira^1^, Frances M Jones^1^, Jakub Wawrzyniak^1^, Colin M Fitzsimmons^1^, Edridah M Tukahebwa^5^, Nicholas Furnham^2,6^, Rick M Maizels^3^ and David W Dunne^1^**

^1.^Department of Pathology, University of Cambridge, UK

^2.^European Bioinformatics Institute, Cambridge, UK

^3.^Institute of Immunology and Infection Research, University of Edinburgh, UK

^4.^Institute of Biological, Environmental and Rural Sciences, Aberystwyth University, UK.

^5.^Vector Control Division, Ugandan Ministry of Health, Uganda

^6.^Department of Pathogen Molecular Biology, London School Hygiene and Tropical Medicine, UK

*** Correspondence:** Edward J Farnell, Department of Pathology, University of Cambridge, Tennis Court Road, Cambridge, CB2 1QE

ef242@cam.ac.uk

**Supplementary Table S3.** Cut off values for antigens determined as mean magnitude plus three standard deviations of responses from 26 uninfected European donors.

| Antigen | IgG1  (μg/ml) | IgG4  (μg/ml) | IgE  (ng/ml) |
| --- | --- | --- | --- |
| SmTAL1 | 45.74 | 0.03 | 0.50 |
| SmTAL2 | 6.55 | 0.011 | 451.80 |
| SmTPM2.8 | 91.50 | 1.88 | 90.90 |
| SmThioredoxin | 0 | 0 | 0 |
| SmCyclophilin | 53.81 | 0.47 | 0 |
| SmTPI | 0 | 0 | 0 |
| SmSOD | 104.00 | 0 | 16.88 |
| SmPGK | 0 | 0 | 0 |
| SmHSP20 | 0 | 0 | 0 |
| SmAldolase | 53.29 | 0 | 0 |
| SmUbiquitin | 0 | 0 | 0 |
| SmDLC | 0 | 0 | 172.12 |
| SmAK | 0 | 0 | 0 |
| SmVAL6 | 7.50 | 0 | 93.26 |
| SmProfilin | 58.30 | 0 | 0 |
| SmLipocalin | 0 | 0 | 0 |
| Sm14-3-3 | 0 | 0 | 0 |
